# Ruptured mediastinal mature teratoma causing severe mediastinitis: report of a surgically resected case and a literature review

**DOI:** 10.1186/s40792-021-01132-8

**Published:** 2021-02-16

**Authors:** Eri Ota, Yujin Kudo, Sachio Maehara, Hideyuki Furumoto, Jun Matsubayashi, Yoshihisa Shimada, Masaru Hagiwara, Toshitaka Nagao, Tatsuo Ohira, Norihiko Ikeda

**Affiliations:** 1 Department of Surgery, Tokyo Medical University, 6-7-1 Nishishinjuku, Shinjuku-ku, Tokyo, 160-0023 Japan; 2 Department of Anatomic Pathology, Tokyo Medical University, 6-7-1 Nishishinjuku, Shinjuku-ku, Tokyo, 160-0023 Japan

**Keywords:** Mediastinal mature teratoma, Rupture, Mediastinitis, Surgical resection

## Abstract

**Background:**

Mediastinal teratomas occasionally rupture into the thoracic cavity, which induces mediastinitis or various other severe complications. Surgical treatment is crucial for ruptured teratomas; however, few literature reviews to date have addressed the characteristics of ruptured mediastinal teratomas.

**Case presentation:**

We report a 29-year-old woman with severe mediastinitis owing to a mediastinal mature teratoma that ruptured into the mediastinum and right pleural cavity. Surgical resection by median sternotomy was performed within 24 hours after emergency admission. Intraoperative findings demonstrated the ruptured wall of the tumor with exposure of its white contents, which appeared similar to skin and fat, and necrotic tissue in the anterior mediastinum. The tumor was adhered to the right upper lobe, the ascending aorta, and pericardium. Owing to the severe adhesion of the tumor caused by inflammation in the surrounding tissues, a small portion of the tumor could not be removed, and hence complete resection with a sufficient surgical margin was not achieved. Pathologically, the tumor consisted of a solid mass and a cystic mass with severe adhesion to the resected portion of the lung, which included skin and lipid tissue. The tumor was concluded to be a mature teratoma as neither an immature component nor malignant transformation was observed. The patient had an uneventful postoperative course.

**Conclusions:**

To our knowledge, this is the report of successful surgical resection of a ruptured mediastinal teratoma causing severe mediastinitis, with the first literature review of ruptured mediastinal teratomas. We also discuss relevant findings from reports in the literature.

## Background

Benign mediastinal germ cell tumors constitute approximately 1.3% (66/5, 197) of all mediastinal tumors ^1^. Mediastinal teratoma is the most common type of mediastinal germ cell tumor, which occasionally ruptures into the thoracic cavity and induces severe complications. Surgical intervention for ruptured teratomas is required in almost all cases. However, there are few reports to date regarding the indications and surgical procedures for the treatment of ruptured teratomas. Herein, we present a surgical case of a patient with a mediastinal mature teratoma that perforated into the mediastinum and thoracic cavity, resulting in severe mediastinitis and pleurisy.

## Case presentation

**Fig. 1:**
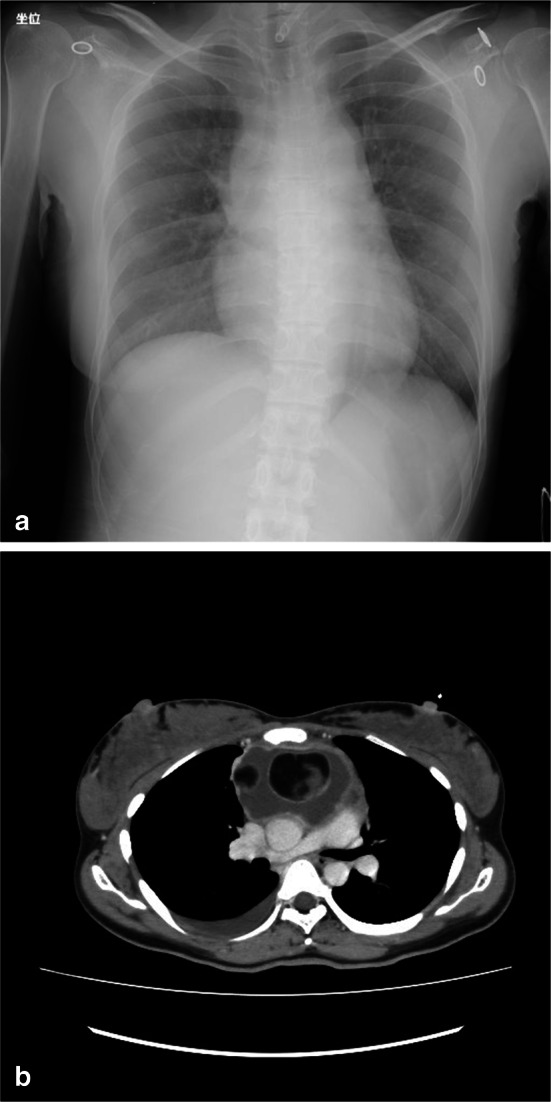
**a** Chest X-ray taken on admission displaying bilateral mediastinal widening. **b** Chest CT displaying a fat-containing cystic mass with solid components in the anterior mediastinum, and pleural effusion in the right side

A 29-year-old woman presented with chest pain that had started 2 months previously. Her right chest pain radiating to the back had suddenly worsened together with the appearance of dyspnea, and she was immediately admitted to our department. On admission, her body temperature, blood pressure, and pulse rate were within the normal range, but her oxygen saturation was 93% at room temperature, and her chest pain continued to worsen. A chest X-ray displayed widening of the upper mediastinum (Fig. 1a). Chest computed tomography (CT) displayed a 9 × 8 × 5 cm heterogeneous cystic mass with an enhanced wall and fat components in the anterior mediastinum (Fig. 1b). Right pleural effusion and fluid collection near the great vessels, including the ascending aorta and the superior vena cava, were also displayed, suggesting tumor rupture into the right thoracic cavity and mediastinum. Her laboratory data demonstrated neutrophilic leukocytosis (white blood cells 10,300/μL; neutrophils: 79.7%) and an increased C-reactive protein level (19.7 mg/dL). The levels of serum α-fetoprotein, human chorionic gonadotropin, and anti-acetylcholine receptor antibody were within the normal range. The patient was diagnosed as having a mediastinal teratoma that had ruptured into the mediastinum and right pleural cavity. Mediastinitis and right pleurisy were also suspected, and therefore an emergency operation was performed just after her admission.

**Fig. 2:**
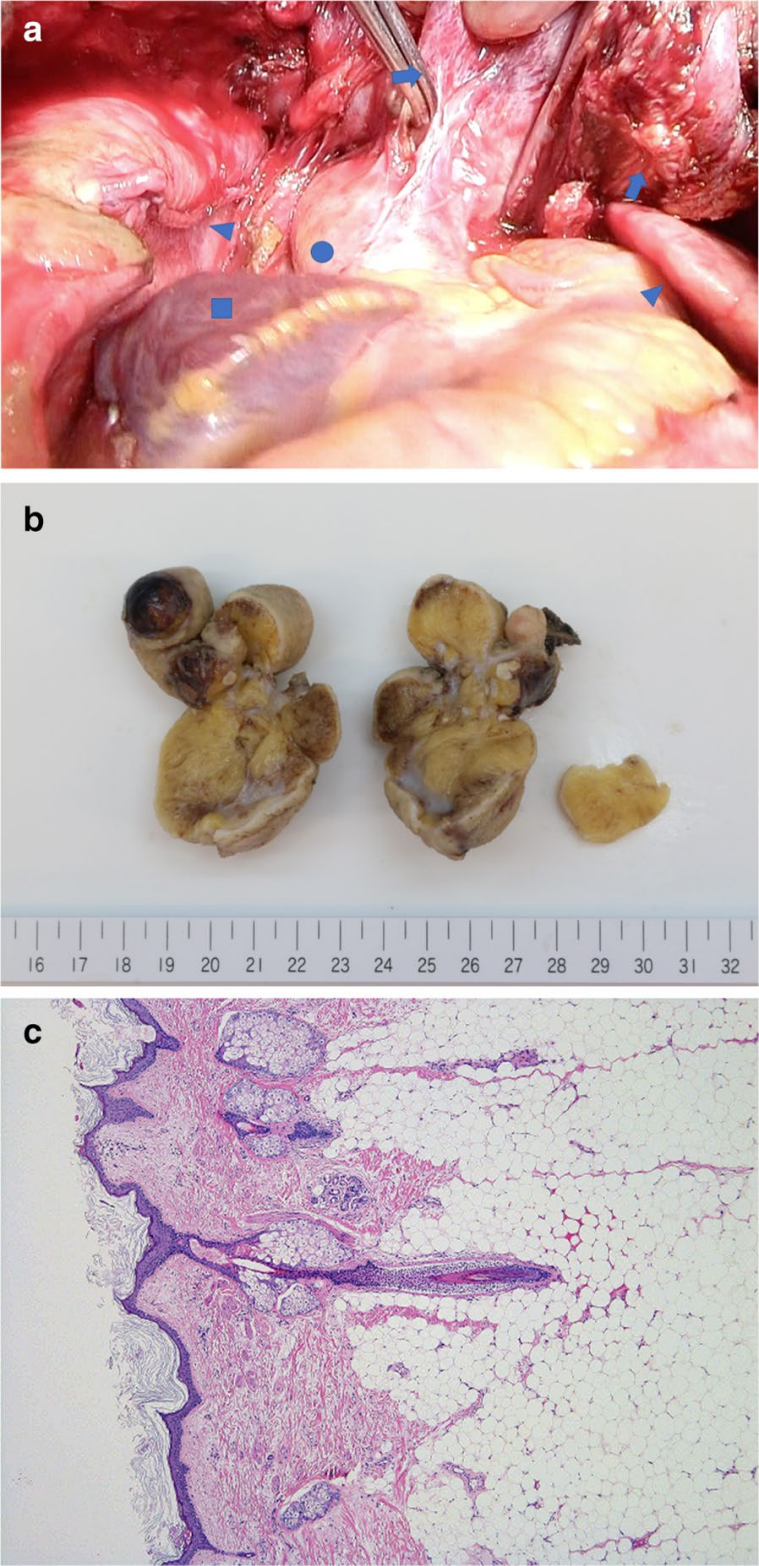
**a** Intraoperative findings showing adhesion of the tumor (arrow) to the ascending aorta (circle) after resection of the pericardium and right upper lung. The bilateral lungs are marked with triangles and the right auricle is marked with a square. **b** The tumor consisted of an 85 × 55 mm solid mass and a 90 × 50 mm cystic mass with severe adhesion to the resected portion of the lung, which included skin and lipid tissue. **c** Hematoxylin–eosin staining of the tumor specimen demonstrating that the inner cavity of the cystic tumor was lined mainly with skin, which was composed of keratinized squamous epithelium, sebaceous and sweat glands, hair follicles, and smooth muscle. The tumor also contained adipose tissue and bronchial elements. The wall of the tumor demonstrated necrotizing inflammation, with the infiltration of many neutrophils, lymphocytes, and macrophages. Neither an immature component nor malignant transformation was observed (low power magnification)

The patient underwent median sternotomy and tumor resection with combined partial resection of the pericardium and right upper lobe. Intraoperative findings demonstrated the ruptured wall of the tumor with exposure of its white contents, which appeared similar to skin and fat, and necrotic tissue in the anterior mediastinum. The tumor was adhered to the right upper lobe, the ascending aorta, and pericardium (Fig. 2a). Pleural effusion with sebaceous fluid was observed in the right pleural cavity. Owing to the severe adhesion of the tumor caused by inflammation in the surrounding tissues, a small portion of the tumor could not be removed, and hence complete resection with a sufficient surgical margin was not achieved. After tumor resection, the mediastinum and the right thoracic cavity were irrigated with a sufficient amount of warm saline to prevent postoperative infection. The chest drainage tubes were placed in the bilateral thoracic cavity and the posterior aspect of the sternum.

Macroscopically, the tumor consisted of an 85 × 55 mm solid mass and a 90 × 50 mm cystic mass with severe adhesion to the resected portion of the lung, which included skin and lipid tissue (Fig. 2b). Microscopically, the inner cavity of the cystic tumor was lined mainly with skin, which was composed of keratinized squamous epithelium, sebaceous and sweat glands, hair follicles, and smooth muscle. The tumor also contained adipose tissue and bronchial elements. The wall of the tumor demonstrated necrotizing inflammation, with the infiltration of many neutrophils, lymphocytes, and macrophages. As neither an immature component nor malignant transformation was observed, the tumor was concluded to be a mature teratoma (Fig. 2c).

The patient had been treated with antibiotics for 7 days from immediately after admission, and the result of the pleural effusion culture was negative. The patient had an uneventful postoperative course and was discharged on postoperative day 11.

## Discussion

Ruptured mediastinal teratomas are relatively rare. There are no standard strategies for their treatment, and physicians make treatment decisions for each individual case. We found that only 56 cases of ruptured mediastinal teratoma have been reported to date, in our literature search of associated or referenced articles for the keywords “mediastinal teratoma” and “rupture” in Pubmed®. To clarify the characteristics of ruptured mediastinal teratoma, we summarized these articles in Table 1, including the types of teratoma, treatment strategies, and timing of surgical intervention (see Additional file 1 for the references). There were 28 women, 21 men, and 7 unknown patients, and the median age was 24 (range: 3–58) years. Chest pain occurred in approximately 60% of the patients, and 40% of them had dyspnea. Leakage of ruptured material from the teratoma into the thoracic cavity was observed in 27%, and cardiac tamponade occurred in 9% of the patients.

**Table 1:** Patient characteristics (*n* = 56)

Variable	Category	*n* (%)
Sex	Men	21 (38%)
	Women	28 (50%)
	Unknown	7 (12%)
Median age (years) (range)		24 (3–58)
Presenting symptoms (duplicated)	Chest pain	35 (63%)
	Dyspnea	21 (38%)
	Cough	12 (18%)
	Fever	12 (18%)
Duration from onset to treatment	< 1 week	13 (23%)
	1–4 weeks	10 (18%)
	4 weeks to 6 months	9 (16%)
	6 months or longer	8 (14%)
	Unknown	16 (29%)
Treatment	Surgery by thoracotomy or median sternotomy	40 (71%)
	Video-assisted thoracic surgery	5 (9%)
	Surgical approach (details unknown)	10 (18%)
	Drainage alone	1 (2%)
Histological type	Mature teratoma	54 (96%)
	Immature teratoma	1 (2%)
	Germ cell tumors with somatic-type solid malignancy	1 (2%)
Prognosis	Survived	44 (79%)
	Died	3 (5%)
	Unknown	9 (16%)
Extramediastinal location of tumor rupture (duplicated)	Lung	16 (29%)
	Thoracic cavity	15 (27%)
	Pericardial sac	7 (13%)
	Unknown	9 (16%)

Severe chest pain occurred in patients when fluid with digestive enzymes or infectious materials from the ruptured tumor spread into the mediastinum or the thoracic cavity and caused inflammation. Necrosis or ischemia caused by tumor swelling also induces tumor rupture. Emergency surgery should be considered in such cases, because the possible resulting mediastinitis and pleurisy have a high risk of causing mortality^2^. Therefore, the early diagnosis and treatment of ruptured mediastinal teratomas are crucial to reduce mortality^3^. However, our literature review demonstrated that surgical resection was performed 1 week after onset of the rupture in 48% of the patients (Table 1). The sudden onset of severe symptoms, such as severe dyspnea, severe chest pain, loss of concentration, and decreased blood pressure, may suggest severe mediastinal inflammation and lead to surgeons deciding to perform surgical intervention in the early phase of tumor rupture.

Surgical resection with a sufficient margin or complete resection is sometimes impossible because of severe adhesion of the tumor owing to inflammation in the surrounding tissue. A preoperative diagnosis to decide on the surgical strategy is important, even though it is difficult to identify the type of teratoma radiologically. Teratomas histologically consist of germ cell tumors with 2 or 3 germ layers, including the ectoderm, endoderm, and mesoderm^4^. They are classified as mature teratomas that comprised mature, adult-type tissues, and immature teratomas comprising embryonic or fetal tissues. Immature teratomas account for only 2% of all mediastinal teratomas, and are considered to be malignant neoplasms, which have the potential to metastasize or recur^5^. Immature teratomas comprise a spectrum of purely mature to predominantly immature tissue from all three germinal layers mixed with embryonic immature tissue. According to our literature review, 2% of patients were found to have malignant ruptured teratoma on postoperative histological analysis, such as an immature teratoma or a germ cell tumor with somatic-type solid malignancy, although most patients were diagnosed as having a mature teratoma (Table 1). In such patients with a malignant teratoma, incomplete resection owing to severe adhesion results in a high risk of recurrence of the malignant disease. Postoperative complications included phrenic nerve paralysis in two patients, pneumothorax in one patient, and recurrent nerve paralysis in one patient.

## Conclusion

We herein reported a case of a patient in whom surgical resection was performed for a ruptured mediastinal mature teratoma that induced mediastinitis, as well as performed the first literature review of ruptured mediastinal teratomas. Our results demonstrate that thoracic surgeons should make appropriate and prompt decisions to treat ruptured mediastinal teratomas and to minimize their associated complications.

## Supplementary Information

**Additional file 1**. The following articles of Supplementary data were summarized in Table 1 to investigate the characteristics of ruptured mediastinal teratomas, including types of teratoma, treatment strategies, and timing of surgical intervention (1-46).

## Data Availability

All data supporting the conclusions of this article are included within the published article.

## Abbreviation

CT: Computed tomography
