# Correction: Ruptured mediastinal mature teratoma causing severe mediastinitis: report of a surgically resected case and a literature review

**DOI:** 10.1186/s40792-023-01697-6

**Published:** 2023-07-18

**Authors:** Eri Ota, Yujin Kudo, Sachio Maehara, Hideyuki Furumoto, Jun Matsubayashi, Yoshihisa Shimada, Masaru Hagiwara, Toshitaka Nagao, Tatsuo Ohira, Norihiko Ikeda

**Affiliations:** 1 Department of Surgery, Tokyo Medical University, 6-7-1 Nishishinjuku, Shinjuku-Ku, Tokyo, 160-0023 Japan; 2 Department of Anatomic Pathology, Tokyo Medical University, 6-7-1 Nishishinjuku, Shinjuku-Ku, Tokyo, 160-0023 Japan

**Correction:** **Surg Case Rep (2021) 7:48. **10.1186/s40792-021-01132-8

Following publication of the original article [1], the name of author Eri Ota has been changed from Eri Nakajima.

The original article [1] has been updated.
