# Supplementary material for: Ruptured mediastinal mature teratoma causing severe mediastinitis: report of a surgically resected case and a literature review
Source: Surg Case Rep. 2021 Feb 16;7:48. doi: 10.1186/s40792-021-01132-8 (PMC7886921; doi:10.1186/s40792-021-01132-8)
Supplement: Supplementary file 1 — Additional file 1. The following articles of Supplementary data were summarized in Table 1 to investigate the characteristics of ruptured mediastinal teratomas, including types of teratoma, treatment strategies, and timing of surgical intervention (1-46). [file 40792_2021_1132_MOESM1_ESM.docx]

**Ruptured mediastinal mature teratoma causing severe mediastinitis: report of a surgically resected case and a literature review**

Eri Ota, MD^1^, Yujin Kudo, MD, PhD^1^, Sachio Maehara, MD, PhD^1^, Hideyuki Furumoto, MD, PhD^1^, Jun Matsubayashi, MD, PhD^2^, Yoshihisa Shimada, MD, PhD^1^, Masaru Hagiwara, MD, PhD^1^, Toshitaka Nagao, MD, PhD^2^, Ohira Tatsuo, MD, PhD^1^, and Norihiko Ikeda, MD, PhD^1^

1. Department of Surgery, Tokyo Medical University, Tokyo, Japan

2. Department of Anatomic Pathology, Tokyo Medical University, Tokyo, Japan

**Supplementary data**

The following articles were summarized in Table 1 to investigate the characteristics of ruptured mediastinal teratomas, including types of teratoma, treatment strategies, and timing of surgical intervention (1-46).

1. Chen C, Zheng H, Jiang S. An unusual case of giant mediastinal teratoma with malignant transformation. Ann Thorac Surg. 2008;86(1):302-4.

2. Agrawal T, Blau AJ, Chwals WJ, Tischler AS. A Unique Case of Mediastinal Teratoma with Mature Pancreatic Tissue, Nesidioblastosis, and Aberrant Islet Differentiation: a Case Report and Literature Review. Endocr Pathol. 2016;27(1):21-4.

3. Yang WM, Chen ML, Lin TS. Traumatic hemothorax resulting from rupture of mediastinal teratoma: a case report. Int Surg. 2005;90(4):241-4.

4. Jung JI, Park SH, Park JG, Lee SH, Lee KY, Hahn ST. Teratoma with malignant transformation in the anterior mediastinum: a case report. Korean J Radiol. 2000;1(3):162-4.

5. Southgate J, Slade PR. Teratodermoid cyst of the mediastinum with pancreatic enzyme secretion. Thorax. 1982;37(6):476-7.

6. Hee HZ, Chen CK, Yeh YC, Huang CS. A symptomatic anterior mediastinal mass with a simultaneous Haemophilus influenzae infection. Respirol Case Rep. 2019;7(2):e00394.

7. Acharya MN, De Robertis F, Popov AF, Anastasiou N. Surgical resection of a huge ruptured mature mediastinal teratoma. Asian Cardiovasc Thorac Ann. 2016;24(7):726-8.

8. Hanten SJ, Keyes TF, Meyer RR. Spontaneous rupture of mediastinal dermoid cysts into the pleural cavity; report of two cases. Radiology. 1955;64(3):348-52.

9. Ege G, Akman H, Cakiroglu G, Kalayci G. Spontaneous rupture of mediastinal cystic teratoma with high levels of amylase, lipase, CA 19-9, CA 125 and CEA in cystic fluid: a case report. Acta Radiol. 2004;45(1):111-2.

10. Matsubara K, Aoki M, Okumura N, Menju T, Nigami H, Harigaya H, et al. Spontaneous rupture of mediastinal cystic teratoma into the pleural cavity: report of two cases and review of the literature. Pediatr Hematol Oncol. 2001;18(3):221-7.

11. Liu CH, Peng YJ, Wang HH, Cheng YL, Chen CW. Spontaneous rupture of a cystic mediastinal teratoma complicated by superior vena cava syndrome. Ann Thorac Surg. 2014;97(2):689-91.

12. Shiota S, Nakaya Y, Sakamoto K, Iwase A, Aoki S, Matsuoka R, et al. Spontaneous hemothorax secondary to immature teratoma of the mediastinum. Intern Med. 1999;38(9):726-8.

13. Inoue Y, Suga A, Yamada S, Iwazaki M. A ruptured mature teratoma in which follow-up computed tomography observation at short intervals was useful for a definitive diagnosis. Interact Cardiovasc Thorac Surg. 2011;12(6):1074-6.

14. Escalon JG, Arkin J, Chaump M, Harkin TJ, Wolf AS, Legasto A. Ruptured anterior mediastinal teratoma with radiologic, pathologic, and bronchoscopic correlation. Clin Imaging. 2015;39(4):689-91.

15. Hiraiwa T, Hayashi T, Kaneda M, Sakai T, Namikawa S, Kusagawa M, et al. Rupture of a benign mediastinal teratoma into the right pleural cavity. Ann Thorac Surg. 1991;51(1):110-2.

16. Kuroda H, Hashidume T, Shimanouchi M, Sakao Y. Resection of a ruptured mature cystic teratoma diagnosed two years after the onset of perforation. World J Surg Oncol. 2014;12:321.

17. Sommerlad BC, Cleland WP, Yong NK. Physiological activity in mediastinal teratomata. Thorax. 1975;30(5):510-5.

18. Tavares JG, Baptista B, Gonçalves B, Raimundo P, Velho HV, Horta AB. Mediastinum Teratoma Rupture: A Rare Manifestation. Eur J Case Rep Intern Med. 2019;6(2):001022.

19. Choi SJ, Lee JS, Song KS, Lim TH. Mediastinal teratoma: CT differentiation of ruptured and unruptured tumors. AJR Am J Roentgenol. 1998;171(3):591-4.

20. Ram D, Sharma DK, Darlong LM, Rajappa SK, Bhakuni YS. Mediastinal Teratoma with Pulmonary Parenchyma Fistula: A Rare Diagnostic Endeavour. J Clin Diagn Res. 2017;11(8):Xd03-xd4.

21. Chen RF, Chang TH, Chang CC, Lee CN. Mediastinal teratoma with pulmonary involvement presenting as massive hemoptysis in 2 patients. Respir Care. 2010;55(8):1094-6.

22. Maillart JF, Lacroix V, Camboni A, Poncelet AJ. Mediastinal teratoma with coexisting parenchymal pulmonary cystic lesion. Ann Thorac Surg. 2013;96(3):1081-3.

23. Liu J, Tian B, Zeng Q, Chen C, Zhou C, Li H, et al. Mediastinal teratoma presenting with hemoptysis and pleuritis misdiagnosed as tuberculosis (empyema). BMC Pediatr. 2018;18(1):382.

24. Suzuki H, Koh E, Hoshino I, Kishi H, Saitoh Y. Mediastinal teratoma complicated with acute mediastinitis. Gen Thorac Cardiovasc Surg. 2010;58(2):105-8.

25. Miyazawa M, Yoshida K, Komatsu K, Kobayashi N, Haba Y. Mediastinal mature teratoma with rupture into pleural cavity due to blunt trauma. Ann Thorac Surg. 2012;93(3):990-2.

26. Yu CW, Hsieh MJ, Hwang KP, Huang CC, Ng SH, Ko SF. Mediastinal mature teratoma with complex rupture into the pleura, lung, and bronchus complicated with mycoplasma pneumonia. J Thorac Cardiovasc Surg. 2007;133(4):1114-5.

27. Serraj M, Lakranbi M, Ghalimi J, Ouadnouni Y, Smahi M. Mediastinal mature teratoma with complex rupture into the lung, bronchus and skin: a case report. World J Surg Oncol. 2013;11:125.

28. Raoufi M, Herrak L, Benali A, Achaachi L, El Ftouh M, Bellarbi S, et al. Mediastinal Mature Teratoma Revealed by Empyema. Case Rep Pulmonol. 2016;2016:7869476.

29. Pikin O, Kolbanov K, Kazakevich V, Korolev A. Mediastinal mature cystic teratoma perforating into the lung. Interact Cardiovasc Thorac Surg. 2010;11(6):827-9.

30. Sanford JR, Clarke D, Hoffenberg R. Mediastinal benign cystic teratoma. J R Soc Med. 1982;75(12):976-7.

31. Ahmed MA, Fouda R, Ammar H, Amin SM. Massive pericardial effusion and multiple pericardial masses due to an anterior mediastinal teratoma rupturing in pericardial sac. BMJ Case Rep. 2012;2012.

32. Omachi N, Kawaguchi T, Shimizu S, Okuma T, Kitaichi M, Atagi S, et al. Life-threatening and Rapidly Growing Teratoma in the Anterior Mediastinum. Intern Med. 2015;54(19):2487-9.

33. Machuca JS, Tejwani D, Niazi M, Diaz-Fuentes G. A large ruptured mediastinal cystic teratoma. J Bronchology Interv Pulmonol. 2010;17(3):269-72.

34. Wheeler D. DERMOID CYST OF THE MEDIASTINUM WITH RUPTURE INTO THE PLEURAL CAVITY. Can Med Assoc J. 1939;41(3):235-6.

35. Cobb CJ, Wynn J, Cobb SR, Duane GB. Cytologic findings in an effusion caused by rupture of a benign cystic teratoma of the mediastinum into a serous cavity. Acta Cytol. 1985;29(6):1015-20.

36. Yang CJ, Cheng YJ, Kang WY, Huang MS, Hwang JJ. A case of dermoid cyst ruptured into the lung. Respirology. 2007;12(6):931-3.

37. Serlo WS, Heikkinen E. Cardiac tamponade caused by a mediastinal teratoma. Scand J Thorac Cardiovasc Surg. 1983;17(3):323-5.

38. Badar F, Yasmeen S, Afroz N, Khan N, Azfar SF. Benign mediastinal teratoma with intrapulmonary and bronchial rupture presenting with recurrent hemoptysis. Iran J Radiol. 2013;10(2):86-9.

39. Maeyama R, Uchiyama A, Tominaga R, Ichimiya H, Kuroiwa K, Tanaka M. Benign mediastinal teratoma complicated by cardiac tamponade: report of a case. Surg Today. 1999;29(11):1206-8.

40. Montebello A, Mizzi A, Cassar PJ, Cassar K. Benign cystic mediastinal teratoma presenting as a massive pleural effusion in a 17-year-old boy. BMJ Case Rep. 2017;2017.

41. Al Smady M, Zahari NNB, Mohd Sahid NSB, Saparudin NSB. Anterior mediastinal teratoma with pericardial effusion. Rare presentation. J Surg Case Rep. 2019;2019(5):rjz136.

42. Jothianandan K, Tibb AS, McLemore M, Keller S, Appel DW. An adult man presenting with hemoptysis caused by mature teratoma with rupture into the bronchus and pericardium and complicated by Haemophilus influenzae infection. J Thorac Cardiovasc Surg. 2010;139(5):e104-7.

43. Thompson DP, Moore TC. Acute thoracic distress in childhood due to spontaneous rupture of a large mediastinal teratoma. J Pediatr Surg. 1969;4(4):416-23.

44. Paterson IM, Cockburn JS. Acute pericarditis due to perforation of a benign mediastinal teratodermoid into the pericardial sac. Thorax. 1982;37(11):863-4.

45. Marsten JL, Cooper AG, Ankeney JL. Acute cardiac tamponade due to perforation of a benign mediastinal teratoma into the pericardial sac. Review of cardiovascular manifestations of mediastinal teratomas. J Thorac Cardiovasc Surg. 1966;51(5):700-7.

46. Suwatanapongched T, Kiatboonsri S, Visessiri Y, Boonkasem S. A 30-year-old woman with intermittent cough and a mass-like opacity in the right upper lobe. Chest. 2011;140(3):808-13.
